# Draft Genome Sequence of *Cercospora arachidicola, *Causal Agent of Early Leaf Spot in Peanuts

**DOI:** 10.1128/genomeA.01281-15

**Published:** 2015-11-05

**Authors:** Valerie A. Orner, Emily G. Cantonwine, Xinye Monica Wang, Amr Abouelleil, James Bochicchio, Chad Nusbaum, Albert K. Culbreath, Zaid Abdo, Renee S. Arias

**Affiliations:** a National Peanut Research Laboratory, Dawson, Georgia, USA; b Department of Biology, Valdosta State University, Valdosta, Georgia, USA; c Broad Institute of MIT and Harvard, Cambridge, Massachusetts, USA; d Department of Plant Pathology, University of Georgia, Tifton, Georgia, USA; e USDA, ARS, SEA, Athens, Georgia, USA

## Abstract

*Cercospora arachidicola*, causal agent of early leaf spot, is an economically important peanut pathogen. Lack of genetic information about this fungus prevents understanding the role that potentially diverse genotypes may have in peanut breeding programs. Here, we report for the first time a draft genome sequence of *C. arachidicola*.

## GENOME ANNOUNCEMENT

Early leaf spot caused by *Cercospora arachidicola* S. Hori (teleomorph *Mycosphaerella arachidis* Deighton) is one of two important leaf spot diseases in peanut (*Arachis hypogaea* L.) responsible for significant economic loss to the industry (1, 2). Infections by *C. arachidicola* appear as small necrotic lesions on the leaves, petioles, or stems, which may be followed by premature defoliation, and, if left unmanaged on susceptible cultivars, can severely decrease yield (1). An effective, yet expensive, disease management strategy consists of multiple fungicide applications throughout the growing season (3). Other strategies such as strip-tillage instead of conventional tillage (4) or weather forecast models that predict disease outbreaks (5) can help minimize the number of fungicide treatments. However, the development of leaf-spot-resistant cultivars that require no fungicide application would be the most desirable means of control (6). The recent completion of the peanut genome (http://www.peanutbase.org) will aid breeding programs, but the negligible amount of *C. arachidicola* genetic information hinders progress. Currently, *C. arachidicola* entries in the NCBI-GenBank database total 8,077 bp in 21 sequences, with half of these entries corresponding to rRNA and the rest only 61 bp each. The genome sequence of *C. arachidicola* will provide relevant information for the advancement of leaf-spot resistant cultivars, be a useful resource to aid in the selection of target genes for disease control, and contribute to the study of genetic diversity of *C. arachidicola*.

A single-spore isolate of *C. arachidicola* from an infected peanut plant near Tifton, Georgia, USA, was grown on potato dextrose agar (Difco, Franklin Lakes, NJ, USA) for 6 months. The fungus was removed from the agar and ground using a Kleco tissue pulverizer (Garcia Machine, Visalia, CA, USA). Genomic DNA was extracted using phenol/chloroform/isoamyl alcohol followed by isopropanol precipitation (7) and cleaned using the GeneJET gel Extraction kit (Thermo, Fisher Scientific, Waltham, MA, USA). Sequencing and assembly were performed by the Broad Institute of MIT and Harvard using an Illumina HiSeq 2500 whole-genome shotgun approach. A total of 465,511,514 reads with an estimated genome coverage of >100× were assembled *de novo* using ALLPATHS (8) and generated 796 contigs (>400 bp each) with an average size of 40,930 bp that assembled into 491 scaffolds (>1,000 bp each). The average size of the scaffolds was 67,710 bp with a total of 33,245,410 bp and a maximum length of 1,387,526 bp. Most hits from BLAST analysis corresponded to the genera *Pseudocercospora* and *Passalora*.

### Nucleotide sequence accession numbers.

This whole-genome shotgun project has been deposited in DDBJ/ENA/GenBank under the accession number LIHB00000000. The version described in this paper is the first version, LIHB01000000.
